# Uncertainty and computational complexity

**DOI:** 10.1098/rstb.2018.0138

**Published:** 2018-12-31

**Authors:** Peter Bossaerts, Nitin Yadav, Carsten Murawski

**Affiliations:** 1 Brain, Mind and Markets Laboratory, Department of Finance, The University of Melbourne, Parkville, Victoria 3010, Australia; 2 Florey Institute of Neuroscience and Mental Health, Parkville, Victoria 3010, Australia

**Keywords:** uncertainty, expected utility, Bayesian, computational complexity

## Abstract

Modern theories of decision-making typically model uncertainty about decision options using the tools of probability theory. This is exemplified by the Savage framework, the most popular framework in decision-making research. There, decision-makers are assumed to choose from among available decision options as if they maximized subjective expected utility, which is given by the utilities of outcomes in different states weighted with subjective beliefs about the occurrence of those states. Beliefs are captured by probabilities and new information is incorporated using Bayes’ Law. The primary concern of the Savage framework is to ensure that decision-makers’ choices are *rational*. Here, we use concepts from computational complexity theory to expose two major weaknesses of the framework. Firstly, we argue that in most situations, subjective utility maximization is computationally intractable, which means that the Savage axioms are implausible. We discuss empirical evidence supporting this claim. Secondly, we argue that there exist many decision situations in which the nature of uncertainty is such that (random) sampling in combination with Bayes’ Law is an ineffective strategy to reduce uncertainty. We discuss several implications of these weaknesses from both an empirical and a normative perspective.

This article is part of the theme issue ‘Risk taking and impulsive behaviour: fundamental discoveries, theoretical perspectives and clinical implications’.

## 1. Introduction

In most modern theories of decision-making, uncertainty about decision options is modelled using the tools of probability theory. This is exemplified by the theory proposed by Leonard Savage in his book *Foundations of Statistics* [1]. In this framework, a decision task is compared to that of a gambler facing a bet in a pure game of chance [2]. Choices are represented by a utility function over state-dependent outcomes and a probability function over states. Decision-makers act as if they maximized (subjective) expected utility.

Importantly, all knowledge about different states of the world—or lack thereof—is represented by probabilities. Probability distributions are usually interpreted as carriers of incomplete information about states of the world [3]. Uncertainty is reduced by (random) sampling and new information is incorporated into probabilities using Bayes’ Law. The framework assumes, at least implicitly, that new knowledge is generated through inference and that such inference is solely concerned with randomness [3]. It is proposed that this way of representing uncertainty is generic, that is, that it can capture any type of uncertainty and learning. All that is needed is that the decision-maker exhibits preferences that are rational, in a way made precise by a set of axioms.

Savage distinguished two important functions of his framework [1,2]. Firstly, it provides a minimal model of rationality. The axioms of the framework are widely considered to be properties that the preferences of any rational decision-maker should have. A decision-maker whose preferences obey those axioms, which are often referred to as the *axioms of rationality*, acts as if she maximized subjective expected utility. Thus, the framework provides a simple decision rule for rational decision-making. Secondly, Savage showed that if preferences of a decision-maker obey his axioms, then their choices can be described by a unique probability measure in combination with a unique (up to affine transformation) utility function. Thus, the framework provides researchers with a theoretically sound and precise toolkit to model choices. The Savage framework is often regarded as a canonical and general framework for the modelling of decisions under uncertainty.

The framework rests on two core assumptions. Firstly, it assumes that a decision-maker always has well-defined preferences in those situations in which the framework is applied [2]. Importantly, it assumes that preferences are transitive and complete. Secondly, it assumes that decision-makers’ beliefs and values can always be disentangled and tracked separately. Another, implicit, assumption requires that the representation of the decision task provided by the framework be appropriate, for example, that the state space underlying beliefs is rich enough to capture the evolution of choices (and hence, preferences) over time. For instance, if the decision-maker chooses differently after a coin toss ends up heads, then the state space should allow for this coin flip.

Few other theories of decision-making have attracted as much attention as Savage’s. In the years after publication, the theory quickly became the dominant framework in decision theory, a status that it has held to this day [3,4]. For decades, the Savage framework has guided empirical research on decision-making under uncertainty. A typical decision task asks the decision-maker to choose from a small number of options that are characterized by pay-offs that depend on a small number of states, which occur with certain probabilities. Pay-offs and probabilities may be provided or have to be learned. A classical task is the Holt–Laury task in which decision-makers have to make a series of choices between a certain pay-off and a gamble in which they may win a reward with probability *p* and nothing otherwise [5,6]. Such tasks have been used widely to characterize utility functions, for example, to measure risk aversion (e.g. [7–9]). They have also been the workhorse tasks to characterize neural signals associated with decision-making [10,11].

Over time, the Savage framework has been criticized on both empirical and theoretical grounds. Important empirical challenges include the behaviours first observed by Allais [12] and Ellsberg [13], both of which demonstrate robust, direct violations of the Savage axioms. Many more deviations have been documented since then [14,15], including evidence that people fail to disentangle values and beliefs in the presence of more than five states [16] and that preferences are not measurement-invariant, for example, that they depend on the way decisions are framed [17], that they depend on the size of the choice set [18,19] and that preferences change with physiological state [20].

Key theoretical challenges have focused on the limits of expressive power of the Savage framework as well as on the plausibility of the axioms from a logical perspective. With regards to the former, the Savage framework requires that knowledge or uncertainty can be expressed in terms of probabilities. This is not always appropriate, for example, because a decision-maker may not have enough information to compute probabilities [2,4]. In addition, the framework is unable to express causal relations, which means that a Savage agent is not able to learn them [21]. Another shortcoming is the inability to represent important logical expressions such as universal quantifiers, which means that expressing certain propositions (e.g. ‘all women are black’) is only possible at the expense of vast amounts of logical resources [4,22].

In relation to logical plausibility of the framework, a core concern has been the potential size of the preference set. In the Savage framework, preferences are defined as binary relations over choice options. For an agent to obey the Savage axioms, the agent needs to have such preferences defined over the entire choice set (all acts available to the agent), an assumption referred to as *completeness of preferences*. However, in many decision situations, this set is extremely large, and it is not clear how an agent would either construct or represent preferences over large sets of options [2,23,24]. Similarly, in order to comply with the Savage axioms, an agent needs to construct a state space that adequately reflects the decision situation. This space, too, might be very large. In addition, when constructing this space, the agent needs to ensure that states are mutually exclusive, which might require a large amount of logical resources, a capability sometimes referred to as *logical omniscience* [22,25]. It has been questioned whether people are able to construct such state spaces in cases where the number of states is large [2,4,23–26].

Many of the theoretical criticisms have been suggestive in nature. For example, while concerns have been expressed that the requirement to have complete preferences or to represent the state space may exceed people’s cognitive capacities, neither the cognitive resources required for such representations nor the cognitive resources available to decision-makers have been quantified. Nor has it been demonstrated empirically that people’s cognitive resources are below those required to obey the Savage axioms.

Two questions arise, related to the two functions of the Savage framework described above. From an empirical standpoint, it needs to be asked whether the Savage framework is an appropriate framework to model human decision-making. This is primarily a question about the plausibility of the framework’s axioms. From a normative perspective, the question is whether it is desirable for an agent to behave as the Savage framework prescribes. Both of these questions have been asked in the past. In this article, we address these questions from a different perspective. We use concepts from computational complexity theory [27–29] to quantify the computational resources required to implement the Savage framework. We show that the framework is *computationally intractable* for both human and machine decision-makers in many, if not most, decision situations, raising doubts about the plausibility of the core axioms of the Savage framework. Subsequently, we show that even in situations in which the Savage framework is computationally tractable, it is *inefficient* in the sense that reduction of uncertainty through (random) sampling is often outperformed by more effective algorithms, questioning the desirability of the Savage framework as a guide to decision-making.

Lack of computational tractability has often been suggested to be of minor concern because Savage proposed an *as if* approach: the decision-maker chooses *as if* there exist utilities over outcomes and beliefs over states, and learns *as if* she uses Bayes’ Law. Concerns about computational plausibility, so the claim, are besides the point since the framework does not describe literally the computations the decision-maker performs. This, however, is a false defense. If a decision-maker’s choices are consistent with a model whose computations are computationally intractable, then the decision-maker’s choices are a solution to a computationally intractable problem. This means that the decision-maker must have solved the problem, which would imply that the problem is not intractable [30].

In addition, if the approach is only *as if* , it loses its normative power, since beliefs and accompanying belief evolution (learning) are entirely subjective. An outsider could not possibly propose a more effective way of learning, for instance, or argue that the beliefs are misguided—one cannot ‘help’ the decision-maker. Thus, the approach can no longer be claimed to provide a foundation for statistical inference [31].

The second issue, related to the *inefficiency* of learning, is even less appreciated. In a probabilistic setting, where laws of large numbers hold, Savage’s approach implies Bayesian learning, and hence, learning is as fast as one can get [32]. This is not necessarily the case in other settings. It is true that Savage’s subjective expected utility theory is generic, and hence, that it applies even in the absence of laws of large numbers. However, it must not be overlooked that the approach assures only consistency (coherence) of choices, and not efficiency in learning. That is, even if one were to follow Savage’s prescription and assign a prior over unknowns, updating this prior based on observed outcomes and Bayes’ Law, it only guarantees that one’s choices are rational and that they will remain rational as evidence accumulates. Learning need not be fast, and certainly not as fast as possible. Interestingly, since statistics is about inference, and Savage’s approach is generally viewed as the foundation of statistics, it is puzzling that it is based on choice coherence and not on learning efficiency! But this does not concern us. Instead, we want to shed light on behaviour of biological organisms under complexity. One could argue there, too, that learning efficiency is key. When an organism needs to adapt fast because survival depends on it, learning speed should probably be given priority over choice consistency. That is, the organism should primarily be concerned with survival, irrespective of whether this involves ‘irrational’ choices.

The remainder of this article is structured as follows. In §2, we briefly recall the key aspects of the Savage framework relevant for our argument. In §3, we introduce two different situations, which we will subsequently use to illustrate tractability and effectiveness of the Savage framework. Then, in §4, we introduce some concepts from computational complexity theory, which we use to assess the computational tractability of the Savage framework. In the subsequent section (§5), we provide empirical evidence that computational complexity theory applies to human decision-making, validating our use of the theory to assess tractability of the Savage framework in the context of human decision-making. Section 6 discusses implications of our findings for decision-making research, from both an empirical and normative perspective.

## 2. The Savage framework

In this section, we will briefly describe the key features of the Savage framework relevant for our argument. At the core of the framework are seven axioms, which are often referred to as the *axioms of rationality*, that is, basic properties that every rational decision-maker should obey. For the purposes of this article, only the first four axioms are relevant. The last three axioms are of a purely technical nature. Therefore, they will not be discussed further. We start with a discussion of the first axiom, establishing *completeness* of preferences, followed by a discussion of the second, third and fourth axioms, establishing the *separability* of belief and value, that is, the agent’s ability to separately track beliefs and values.

### (a) Completeness of preferences

Savage’s basic setting is a situation in which an agent is faced with the choice of an act from a set *F*_0_ of possible acts, the consequences of which are uncertain. We start with defining a set of possibilities, which we will refer to as the set of *states of the world* or *propositions* *S*. This set, which is sometimes also referred to as *possible worlds* or *elementary outcomes*, contains the descriptions of the ways in which unknowns may turn out. It is assumed that the elements of *S* are mutually exclusive and collectively exhaustive. A set of states is referred to as *event*.

We also define the set *C* of consequences, which contains descriptions of the ways in which the consequences of the choice of a particular act may turn out. The elements of *C* are also assumed to be mutually exclusive and collectively exhaustive.

For each element *s* in *S* and each act *f* in *F*_0_, let *f*(*s*) describe the element of *C* that describes the consequences of act *f* if state *s* is the correct description of the decision-maker’s situation. Each act in *F*_0_ describes a mapping from *S* to *C*. We denote by *F* the set of all mappings from *S* to *C*. We will call all elements of *F* *acts* and the elements of *F*_0_ *concrete acts*.

Next, we introduce a binary relation ≾ on the acts in *F*, which we interpret as ‘is less preferred’. That is, when faced with a decision between acts *f* and *g*, f≾g will mean that the agent either prefers *g* to *f* or is indifferent between the two.

Savage’s first axiom, or postulate, assumes that agents rank all acts available to them. More precisely, it is assumed that the relation ≾ is a total preorder, that is, that it is both transitive and total (and hence reflexive). Transitivity means that, for all acts *f*, *g* and *h* in *F*, if f≾g and g≾h, then f≾h—that is, if the agent prefers *g* to *f* and *h* to *g*, then it will also prefer *h* to *f*. Totality means that for all *f* and *g*, we have either f≾g or g≾f—that is, for all acts available to the agent, it either prefers *f* to *g* or *g* to *f*. This implies reflexivity, that is, for all *f*, f≾f—the agent cannot strictly prefer *f* to itself. If we have both f≾g and g≾f, we say that the agent is indifferent between *f* and *g*.

The plausibility of the completeness axiom is often illustrated with relatively simple decision situations. Indeed, if a person is given the choice between an apple, an orange and a banana, it seems reasonable to assume that the person is able to rank all options and that this ranking will be transitive—for example, that the person prefers an apple to an orange, an orange to a banana and an apple to a banana.

Consider a different setting. Suppose a person is given the choice between subsets of goods from a set of 10 available goods, subject to a budget constraint. The number of subsets that can be formed from 10 goods is 1024 (2^10^). For the completeness axiom to be satisfied, that is, for the person to establish a total preorder over all possible acts, they would have to check the budget constraints for all 1024 sets and make up to 102 247 563 binary comparisons ($\sum_{k=0}^{10}k!S(10,k$) where *S* is the Sterling number of the second kind). For reference, the number of unique items in a typical American supermarket exceeds 40 000.

Very quickly after Savage first proposed his framework, doubts were raised over the validity of the completeness axiom (e.g. [23]). A discussion ensued whether Savage assumed that preferences exist before a person is put into a decision situation (determined ‘offline’) or whether preferences are constructed in the decision situation (determined ‘online’) [2]. In case of the former, it is not clear how a decision-maker would store preference sets for all decision situations encountered in every-day life, particularly given their size. In case of the latter, the question arises why a decision-maker would construct preferences over all decision options, as is assumed in the completeness axiom, including all options that are not chosen. In any case, it appears unlikely that a decision-maker always has complete preferences over all acts, pre-existing or constructed, given that this would require a number of binary comparisons that would often exceed the number of neurons in the brain (about 8.6 × 10^10^, [33]; cf. [30,34]).

The situation becomes more complicated in a dynamic setting in which new information arrives continuously. Remember that the primitives in Savage’s framework are preferences and not probabilities or utilities. Indeed, the latter are deduced from preferences. Thus, the Savage framework is radically behaviourist in its approach, which is in stark contrast with many modern approaches in cognitive science and artificial intelligence [4]. Savage justified his choice arguing that preferences could be directly observed while probabilities and utilities could not [1,2]. This means, however, that whenever new information becomes available about any of the states, the agent needs to reconstruct the entire preference set, which implies, and from which, a new probability measure can be deduced.

In §4, we will use computational complexity theory to quantify the computational resource requirements that implementation of the completeness axiom would require. We will argue that these requirements quickly exceed the cognitive capacities of human decision-makers—as well as the resources of the world’s most powerful supercomputers—and we will present empirical evidence suggesting that the completeness axiom is indeed implausible in most decision situations, for both human and machine decision-makers.

### (b) Dissociation of belief and value

The second, third and fourth axioms of the Savage framework are necessary to ensure that the decision-makers’ beliefs and values can always be disentangled, the second core implication of the framework. Together, these axioms assume that agents can track beliefs and values separately. For Savage, the ability to disentangle beliefs and values was central to the concept of rationality [1]. Our exposition of these axioms follows Shafer [2], which is more intuitive than the original exposition in Savage [1].

To discuss these axioms, we need to introduce some additional notation. For each act *f* in *F* and each subset *A* of *S*, we denote by *f*_*A*_ the restriction of the mapping *f* to the set *A*. A subset *A* of *S* is called *null* if *f* ∼ *g* whenever *f* and *g* are elements of *F* such that $f_{A^{c}}∼g_{A^{c}}$, where *A*^c^ denotes the complement of *A*. This condition means that the agent’s preferences among acts are not influenced by the consequences they have for states in *A*. The subset *A* is called *null* in this case because it is presumed that the agent’s indifference towards *A* indicates the belief that the true state of the world is not in *A*.

Let *A* be a subset of *S* and *p* and *q* be two mappings from *A* to *C*. We write p≺q if f≺g for every pair *f* and *g* of mappings in *F* such that *f*_*A*_ ∼ *p*, *g*_*A*_ ∼ *q* and $f_{A^{c}}∼g_{A^{c}}$. We denote by [*c*] the act in *F* that maps all *s* in *S* to *c*, for a given *c* in *C*. We call such an act a *constant act*. We are now ready to state the remaining axioms postulated by Savage.

Savage’s second axiom, often referred to as the *sure-thing principle*, states that if f≺g and $f_{A^{c}}∼g_{A^{c}}$, then $f_{A}≺g_{A}$. The axiom says that if two acts agree on *A*^c^, then the choice between them should only depend on how they differ on *A* but not on how they agree on *A*^*c*^. This axiom has been the most controversial of the Savage axioms [2]. Many empirical studies have demonstrated that people robustly violate the axiom [14,15]. The most famous examples of such violations are the Allais and the Ellsberg paradoxes [12,13].

The third axiom ensures that value can be separated from belief. Formally, if *A* is not null, then ${\left[c\right]}_{A}≺{\left[d\right]}_{A}$ if and only if [c]≺[d]. This axiom says that if an agent prefers *d* to *c* in general, then it prefers it in every event *A*. Specifically, if *A* consists of a single state *s*, it means that the agent prefers *d* to *c* in every state of the world; that is, the state of the world is irrelevant for preference—preferences are independent of belief.

The fourth axiom assumes that belief can be discovered from preference. Suppose [c]≺[d], *f* is equal to *c* on *A* and *d* on *A*^c^, and *g* is equal to *c* on *B* and *d* on *B*^c^. Suppose, similarly, that $\left[c^{\prime }]≺[d^{\prime }\right]$, *f*′ is equal to *c*′ on *A* and *d*′ on *A*^c^, and *g*′ is equal to *c*′ on *B* and *d*′ on *B*^c^. Then f≺g if and only if $f^{\prime }≺g^{\prime }$. This axiom means that value is independent of belief, that is, that the preference for *d* over *c* is independent of whether the true state of the world is in *A* or in *B*. Then the only available explanation for the preference f≺g is that the agent considers *B* more likely than *A*. For this to hold, though, the preference f≺g must remain the same when *c* and *d* are replaced by any other pair of consequences *c*′ and *d*′ such that $\left[c^{\prime }]≺[d^{\prime }\right]$. This is what the fourth axiom posits.

Many criticisms have been raised against the third and fourth axioms [2]. Perhaps the most serious one is the implicit assumption that the agent constructs the state space and set of consequences in a way that is suitable for the decision task at hand. The two sets are interdependent and all states in the state space as well as all consequences in the set of consequences need to be mutually exclusive and collectively exhaustive in order to comply with the assumptions of the framework, a task that quickly demands enormous logical resources [25]. Moreover, the state space needs to be refined enough to capture all decision-relevant aspects of uncertainty. In most decision situations, it is not obvious how to set up the state space and it is even less obvious how a decision-maker would always design the state space in such a way that is optimal to solve the decision task at hand. If, on the other hand, the design of the state space was made part of the decision task, then the agent could easily end up in an infinite regress [2].

### (c) Representation

The Savage axioms imply the existence of a probability measure *μ* on *S* and a real-valued function *U* on *C* such that f≺g if and only if *E*_*μ*_[*U*(*f*)] < *E*_*μ*_[*U*(*g*)]—that is, an agent’s preferences among acts can be represented by subjective expected utility. It means the weighted average of utilities of outcomes for *f* is less that of *g*, with the weights provided by the decision-maker’s beliefs that the states occur. The axioms thus imply that an agent’s behaviour can be represented in a way that appears *as if* the agent maximized subjective expected utility. Details of the proof of this result are not relevant for our argument. The interested reader is referred to Savage [1].

As indicated in the Introduction, the Savage framework can serve both an empirical and a normative function. Regarding the former, if it can be assumed that the preferences of a decision-maker obey the rationality axioms, then choices can be described by a unique probability measure in combination with a unique (up to affine transformation) utility function. Thus, the framework provides a theoretically sound and precise toolkit to model choices. In the following, we will primarily be concerned with this function of the Savage framework.

## 3. Two examples

We now present two examples of canonical decision situations. The first example will look familiar to decision-making researchers, while the second example has a structure not commonly encountered in decision science. We will use these examples to expose some of the limits of the Savage framework.

### (a) Selection of securities

In the first situation, a decision-maker is faced with a choice to select securities from a set of 10 securities to maximize her subjective expected utility. Each security eventually pays off either $ 1 dollar or nothing. The type of each security (pay-off) is determined by chance with probability 0.5. The decision-maker can collect information over 30 periods to learn the types prior to selecting the set of securities. In each period (trial), the decision-maker receives a signal from each of the securities, which is positive with probability 0.7 and negative with probability 0.3 if the final dividend of the security is 1, and positive with probability 0.3 and negative with probability 0.7 otherwise.

Application of the Savage framework to this situation is straightforward. Given that there are 10 securities and each pays off either $ 1 or $ 0, these 10 securities collectively can be in 1024 (2^10^) different states in each period, or 2^300^ after 30 periods. Each of those states is one possible combination of pay-offs, for example, all 10 securities pay $ 0. The set of consequences would be the dollar amount received in each of the states, ranging from $ 0 to $ 10, depending on the subset of securities chosen. As the decision-maker has to choose three securities from the ten available, the set of acts are all the mappings from the state space to the set of consequences, for each of the different available choices of three securities.

Each time the decision-maker receives a signal from each of the securities, she uses Bayes’ Law to update her beliefs about the final pay-offs, given by a probability measure over the state space. Assuming a quadratic loss function, her initial valuation of the securities equals the posterior mean, starting from an unconditional estimate of 0.5.

Importantly, the decision-maker has to express preferences over all possible states, since it is preferences (choices) that reveal her beliefs, and the evolution of her beliefs (e.g. she could imagine that the signals are dependent across securities, or over time). As we pointed out in the previous section, we can only observe preferences (choices) but not beliefs, which means that we would have to deduce beliefs from observed preferences.

### (b) Backpacking

In the second example, the decision-maker is faced with the following task. There are 10 items, with the following values (first number) and weights (second number): (31, 21), (141, 97), (46, 32), (30, 21), (74, 52), (105, 75), (119, 86), (160, 116), (59, 43), (71, 54). The decision-maker has to find a subset of items from the 10 items available with the maximum total value but whose total weight does not exceed 265. This is an instance of the 0–1 knapsack problem [35]. We also assume that there is a security for each available item, that is, there are 10 securities in total, one corresponding to each item. If an item is in the optimal solution, then the corresponding security’s pay-off will be $ 1; otherwise, it will expire worthless.

As in the previous example, the decision-maker can select securities from a set of 10 securities and does so with the goal to maximize her subjective expected utility. We assume that the decision-maker can try out (sample) different combinations of items before choosing the securities. Each time she receives a sample, she updates her beliefs using Bayes’ Law.

In this example, we can set up the state space in the same way we did in the previous example. Each state is one possible combination of pay-offs, and the set of consequences would be the dollar amount received in each of the states, ranging from $ 0 to $ 10. And like in the previous example, the set of acts are all the mappings from the state space to the set of consequences, for each of the different available choices of three securities.

How would the decision-maker apply Bayes’ Law to the samples in this situation? The decision-maker could assign a prior probability to each security, which can be completely arbitrary. For example, she could assign the value 0.5 to each security, which could be interpreted as the belief that each item has 50% chance of being part of the solution. She could then gradually adjust her beliefs by sampling, as follows. In each ‘sample’, she randomly picks a subset of items until she reaches the weight limit. She then computes the value of this subset and compares it to the maximum value obtained in previous samples. She then constructs ‘signals’ of the likelihood that an item is in the optimal solution, as follows. If the new value of the subset is less than the previous maximal value, then the signal for an item equals 1 if it was in the earlier (better) solution, and 0 otherwise. If the new total value is higher than the trailing maximum, then the signal equals 1 if the item is in the current solution; otherwise, the signal is 0. The decision-maker then updates her beliefs in each sample *t* (*t* = 1, …, *T*) by weighing the belief in the previous trial (*t* − 1) by (*t* − 1)/*t* and the signal in the present trial by 1/*t*. This may not appear to be in accordance with Bayes’ Law because it does not use the true likelihoods (of observing a particular subset value given the optimal solution). However, this is without consequence, because Savage only requires the decision-maker to use some prior, not the true distribution. That is, our decision-maker is allowed to start with an incorrect set of beliefs. Indeed, as we will show below, it is unreasonable to assume that the decision-maker knows the true likelihoods as this would require for the decision-maker to know the solution of the problem.

At the outset, these two examples may look very similar. In both tasks, the decision-maker tries to maximize subjective expected utility by selecting a set of securities from 10 securities available. Yet, as we will show below, the two decision tasks are fundamentally different in terms of their mathematical properties. While it may appear that the Savage framework can be applied to both tasks, we will show that the framework is computationally intractable in the second type of task, and that it is ineffective for such tasks. To demonstrate that this is indeed the case, we now introduce some key concepts from computational complexity theory.

## 4. Computational complexity

The Savage framework assumes that decision-makers act in a way that is consistent with the axioms of rationality described in §2. In particular, it requires that the decision-maker always choose the most preferred act from all acts available, which means that the decision-maker needs to determine the most preferred act. The latter implies a computational problem. Our concern in this section is to determine the computational resources required to solve such problems.

Computation can occur in multiple physical and formal systems. To study properties of computation (e.g. its complexity), Alan Turing provided an elegant mathematical model called the *Turing machine*, which is capable of simulating *all* types of physically realizable computation [36,37]. It is widely believed that Turing machines can also simulate the human brain—a notion captured by the so-called *Church-Turing thesis*. Computation generally involves reading an input (e.g. the values and weights of all available items in the backpacking example in §3) and producing an output (e.g. 1 if an item is in the optimal solution and 0 otherwise). The number of computational steps required is often represented as a function of the size of the input. Computational efficiency of an algorithm can be measured based on how the number of atomic computational steps (i.e. compute time) scales as a function of input size.

A *complexity class* is a set of computational problems whose solution can be computed within certain resource bounds [29]. Complexity classes have typically been studied in the context of decision problems, that is, computational problem with a yes/no answer. However, the analysis can be extended to more general types of problems.

A (decision) problem is in the complexity class **P** if all instances of size *n* of this problem can be solved by an algorithm that takes at most *kn*^*c*^ computational steps (time), for some constants *k* and *c*. Informally, this means that for a problem in complexity class **P** with input size *n*, one may need to wait an amount of time—in the worst case—that is polynomial in *n*. Problems in class **P** are often called *tractable* since they can be solved efficiently, that is, with a reasonable amount of resources.

In contrast to problems that can be efficiently solved, the complexity class **NP** contains problems whose solutions can be efficiently *verified*. Many important computational problems are in class **NP**, including the travelling salesperson problem (decision version), the satisfiability problem, the set cover problem and the 0–1 knapsack problem (decision version). At present, it is an open question whether **NP** is a strict superset of **P**, known as the ***P** versus **NP** problem* [38].

The second example in §3 is an optimization problem (the optimization version of the 0–1 knapsack problem): its answer is the solution of the problem, here, the value of the optimal knapsack. Optimization problems generally consist of two parts: (i) *feasibility* conditions (e.g. the sum of weights of items in a knapsack must not exceed a given constraint) and (ii) one variable to optimize (e.g. the sum of values of the items in the knapsack).

A decision variant of an optimization problem can be solved by first computing the optimum of the optimization problem, and then checking the optimal value against its decision threshold. Hence, if one could solve the optimization variant efficiently, it would imply that the decision variant can also be solved efficiently. Or, in other words, the optimization problem is at least as hard as its decision variant. The complexity class **NP-hard** contains problems that are as at least as hard as the hardest problems in the class **NP**. The optimization version of the 0–1 knapsack problem is **NP-hard**. The amount of computational resources (time, memory) required to solve them quickly becomes astronomical.

As the definitions indicate, complexity classes such as **P** and **NP** are defined in terms of the asymptotic worst-case use of a computational resource, such as time, as a function of the size of the problem’s input. However, for a given size of a problem’s input, the use of resources may vary substantially. For example, sorting an array of the same set of numbers—that is, the same input length—that is already in the desired order will tend to take less time than sorting an array that is completely out of order (depending on the algorithm used).

We call a particular input to a computational problem an *instance* of the problem. For example, a particular array of numbers is an instance of the sorting problem. The second example in the previous section is an instance of the 0–1 knapsack problem. To analyze the computational resource requirements of decision tasks, it is important to know which computational resources are required for the instances of computational problems encountered by decision-makers. For example, decision-makers may never encounter the worst case of the 0–1 knapsack problem and therefore the worst-case behaviour of solution algorithms for this problem may be irrelevant for every-day decision-making.

It has been shown that some intractable (**NP-hard**) problems become tractable (solvable in polynomial time) if the input is restricted to certain instances. More precisely, for these problems compute time scales super-polynomially with input size. However, compute time only scales polynomially with certain other parameters of the input. This means that these problems can be solved in polynomial time, that is, are tractable, for certain values of input parameters [30,39].

But even in this kind of analysis, referred to as *parametrized complexity*, the focus is on the worst-case behaviour of resource requirements. Researchers have started to characterize the computational complexity of individual instances of computational problems, with the aim to link properties of individual instances to computational resource requirements [40–45]. An important set of results has been able to achieve this goal for a number of important computational problems in the class **NP-complete**, the class of the hardest problems in **NP**, including the decision version of travelling salesperson problem [41], the satisfiability problem [42] and the (decision version of) the 0–1 knapsack problem [46]. For these problems, researchers have shown that the instances with the highest computational resource requirements tend to lie in a narrow range of values of certain instance parameters. At this point, little is known about what the parameter values are of instances typically encountered in real life. In those cases that have been studied, it has been shown that many real-world instances have high degrees of complexity [41,47].

These results on the computational complexity of instances do not rely on a particular computational model or algorithm. The fact that it has been possible to relate computational resource requirements of instances to particular properties of instances indicates that computational complexity may be an inherent feature of instances and, by extension, classes of computational problems, as opposed to a feature of a particular computational model [46].

## 5. Computational complexity and human decision-making

In this section, we briefly review empirical evidence suggesting that computational complexity, as quantified by computational complexity theory, affects human decision-making. This is not immediately obvious. Absent a formal description of the principles of computation in the human brain, it is basically an open empirical question. Assuming that people’s computational resources, in particular time (energy) and memory are limited, we would expect that people can only solve those instances of computational problems whose computational resource requirements are below the resource limits and that human ability to solve instances decreases as computational complexity increases [34].

**Figure 1.**
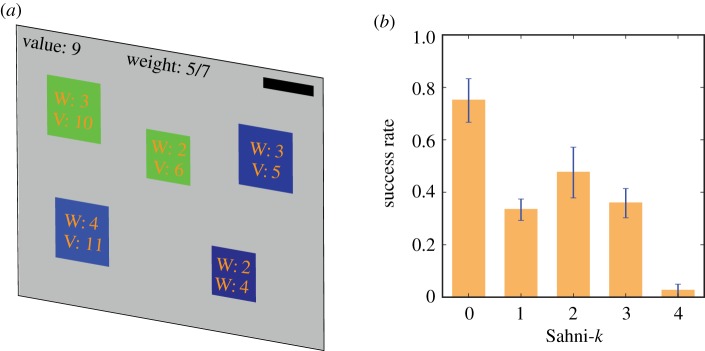
Human performance in the 0–1 knapsack problem. (*a*) Task interface. In this example, there are five items that differ in their values and weights. The goal is to find the subset of available items that maximizes total value of the knapsack, subject to a capacity constraint of 7. In this relatively simple example, the optimal solution contains items 1 and 3. (*b*) Mean success rates across attempts of instances, stratified by Sahni-*k* level. Blue lines, standard errors of means. (Reproduced from Murawski and Bossaerts [35].) (Online version in colour.)

In one study, participants were presented with a set of instances of the 0–1 knapsack problem, the same problem as in the second example in §3 [35]. In each instance, participants had to find from a number of items—between 10 and 12—the subset with the maximum total value subject to a total weight constraint. To do so, people could try out different sets of items on a computer interface, which automatically computed total value and weight of the items selected (figure 1*a*). Participants were given 5 min to solve an instance, but could submit a solution as soon as they thought they had found the solution. However, participants almost never exhausted the time limit. Participants were rewarded based on the proximity of their suggested solution to the optimal solution of an instance.

To quantify the computational complexity of each instance, the Sahni-*k* metric was computed. The metric is proportional to the number of computational operations and the amount of memory required to solve an instance. It is based on the Sahni algorithm, an algorithm designed to solve the 0–1 knapsack problem [48]. Intuitively, the Sahni algorithm works as follows. Firstly, it computes all subsets of items of cardinality *k* that can be formed from the set of available items. Then it considers each of these subsets and uses the greedy algorithm to fill the remainder of the knapsack to capacity. The greedy algorithm selects items in the reverse order of their value-to-weight ratio and terminates once the knapsack has reached capacity. That is, if the Sahni algorithm is executed with *k* equal to 0, it uses the greedy algorithm to fill the knapsack. This is the algorithm’s proposed solution. If *k* is equal to 1, then the algorithm considers subsets of cardinality 1, one for each of the items available, and extends these subsets using the greedy algorithm. The proposed solution is the subset with the highest total value. Instances of the 0–1 knapsack problem differ in the value of *k* required for the algorithm’s candidate solution to be the instance’s actual solution. The higher the value of *k*, the higher the computational complexity of instances, because the higher the computational resource requirements necessary to solve the instance.

If human behaviour was affected by computational complexity, then we would expect a participant’s ability to find the solution of an instance to decrease as the value of Sahni-*k* increases. This is exactly what was found [35]. While for those instances with Sahni-*k* equal to 0, about 77% of participants found the instances’ solutions, this proportion dropped to about 7% for instances for which Sahni-*k* was equal to 4 (figure 1*b*). This result demonstrates that participants’ ability to solve instances of the 0–1 knapsack problem was affected substantially by computational complexity. The study replicated findings of an earlier study that had used the same set of instances of the 0–1 knapsack problem [49].

The study also considered effort extended by participants on instances. Participants extended more effort on instances with higher levels of Sahni-*k*, that is, higher levels of computational complexity. This means that participants adjusted their effort to the level of computational complexity, which could be interpreted as if participants allocated more computational resources to the decision task when the level of computational complexity was higher. Nevertheless, participants’ ability to solve instances decreased with increasing computational complexity. This suggests that the computational resource requirements on instances with higher levels of computational complexity exceeded the resources available.

Similar results have since been obtained in other studies. For example, it has recently been shown that the ability of human participants to find the optimal solution to the set cover and maximum coverage problems can be predicted from a set of mathematical properties of the graph representations of the problem instances [50]. This study used structural properties of instances to quantify computational complexity, as opposed to algorithm-based measures, and confirmed that people’s ability to solve computational problems decreased with increasing levels of computational complexity.

Together, these results strongly suggest that computational complexity affects human decision-making, that is, human ability to solve computational problems decreases with increasing levels of computational complexity [34]. This, in turn, means that we can apply computational complexity theory to the Savage framework to assess the framework’s tractability and, thus, the plausibility of its axioms.

## 6. Limits of the Savage framework

In this section, we discuss the implications of computational complexity for human decision-making theory, in particular, for the Savage framework. We focus on two key issues. Firstly, we will ask whether the assumptions of the Savage framework are plausible, given the computational complexity of the computational problems decision-makers have to solve in order for their behaviour to be compliant with the Savage axioms. To this end, we will investigate whether decision tasks implied by the Savage framework are computationally tractable. Secondly, we will ask whether the Savage framework is effective in representing the type of uncertainty encountered in decision tasks with high computational complexity such as the second example in §3.

### (a) Tractability

To assess the computational tractability of the Savage framework, we first consider the completeness axiom (see §2). This axiom requires that decision-makers have preferences that are both transitive and complete. This, however, means that in any decision situation, they make binary comparisons of all acts available to them. While the number of such comparisons may be small in some decision situations, in many decision tasks it quickly becomes astronomical (see §4). We can therefore assume that in most decision situations, it would be implausible to presuppose that decision-makers have, or are able to construct, a transitive and complete set of preferences over all acts.

One might argue that in order to identify the most preferred act, decision-makers use strategies that do not involve a comparison of all available acts. However, many decision tasks, in particular in economic decision-making, have a structure similar to that of the knapsack problem, have the knapsack problem as a subproblem or have similar levels of computational complexity [34]. The knapsack problem is **NP-hard**, which means that there is no known algorithm that (i) is guaranteed to always find the solution of an instance, and (ii) runs in polynomial time. While human decision-makers may use many different strategies, which may differ from computer algorithms, it is unreasonable to assume that they would overcome the computational complexity of the problem.

This means that many decision tasks are computationally intractable. In the light of the studies discussed in §5, it is therefore unreasonable to assume that people are able to solve them, which would require somehow identifying the most preferred, that is, the highest ranked act. It has been shown that rational choice in the general case is not computable [24].

A second, related issue arises in relation to beliefs in the Savage framework. As discussed in §2, the framework assumes that decision-makers have a set of beliefs over all states of the world and that they update these beliefs in a way that is consistent with Bayes’ Law. Remember, however, that the primitive in the framework are preferences rather than beliefs (and values). Beliefs (and values) are only used to represent preferences. Therefore, beliefs—and changes of beliefs—have to be inferred from (changes of) preferences.

Two issues arise. Firstly, if completeness of preferences is intractable, the completeness axiom is violated and the representation theorem no longer holds. This, in turn, means that inference of beliefs from preferences may no longer be possible.

Secondly, for the completeness axiom to be obeyed dynamically, the decision-maker would have to update her beliefs in accordance with Bayes’ Law whenever new information becomes available. Technically, this means that the decision-maker would have to change her preferences so that they remain consistent with Bayes’ Law. It has been shown that some of the computational problems involved in such updating are computationally intractable [51]. Note also that these preferences effectively have to be determined dynamically over time. In the first example in §3, preferences would have to be complete over 2^300^ possible binary choices.

It has sometimes been argued that people do not literally implement the subjective expected utility maximization but that their behaviour closely approximates it. Technically, this would mean that decision-makers are somehow able to approximate the optimal solution of the computational problem implied by the Savage framework. In computer science, an approximation algorithm is an algorithm that is inexact in the sense that it is not guaranteed to find the optimal solution but is guaranteed to closely approximate it. It has been shown that many computational problems do not allow for approximation, in the sense just defined, in polynomial time [29,30,52]. That is, it is often as hard to compute an approximate solution as it is to compute the value of an optimal solution. Therefore, it is implausible to expect decision-makers to always be able to approximate subjective expected utility maximization, as this would at least sometimes require them to solve intractable problems.

We conclude that the Savage framework is computationally intractable not only from the perspective of completeness, but also from the perspective of maintaining a set of beliefs consistent with Bayes’ Law.

### (b) Effectiveness

We now turn to a different question, asking whether the Savage framework is an effective way to represent decision situations, in particular, whether it is effective in representing the kind of uncertainty encountered in decision situations. To this end, we will draw on the two examples introduced in §3.

**Figure 2.**
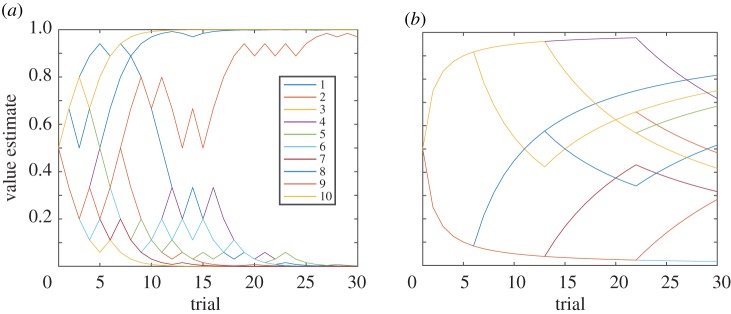
Reduction of uncertainty through (random) sampling. (*a*) Evolution of beliefs for each of 10 securities over 30 trials in the securities selection example (first example in §3). The final payoffs of the securities are 0, 0, 0, 0, 0, 0, 0, 1, 1 and 1 for securities 1–10, respectively. (*b*) Evolution of beliefs for each of 10 securities over 30 trials in the backpacking example (second example in §3). Items 2, 5 and 8 are in the optimal solution and corresponding securities pay $1. All other securities pay zero.

In the first example, the decision-maker is presented with the task of selecting three securities with probabilistic pay-offs. Probabilities can be learned by sampling. Using Bayes’ Law to update beliefs means that beliefs quickly converge to the underlying (true) probabilities (figure 2*a*). Notice how valuations quickly separate between those that will end up paying $ 1 and those that expire worthless. The final payoffs of the securities are 0, 0, 0, 0, 0, 0, 0, 1, 1 and 1 for securities 1 to 10, respectively. As such, using repeated sampling, beliefs quickly converge to the (true) probabilities. As time progresses, the chance of a valuation veering off (say, from close to 1 down to 0) is drastically reduced. Even with a finite (and small number of) samples (signals), the valuation estimate is *probably approximately correct* [53,54]. Indeed, it can be shown that in this situation, updating beliefs using Bayes’ Law is not only rational but also the optimal way of processing information [32]. In this particular example, belief updating is tractable (in class **P**). Note, however, that in general, computing probability distributions both exactly and approximately is **NP-hard** [55,56].

We now turn to the second example in §3. In this example, uncertainty is of a different nature. It is not probabilistic but arises from computational complexity: computing the optimal solution requires a high degree of computational resources.

As we described in §3, a decision-maker could still use the Savage framework to represent uncertainty in this example. Probabilities would represent the degree of belief that a certain subset of items is the optimal solution. Information gained by trying out another randomly chosen possible combination of items could be integrated into beliefs using Bayes’ Law, as described in §3. In the example, items 2, 5 and 8 are in the optimal solution and, hence, the corresponding securities end up paying $ 1; all others expire worthless (figure 2*b*).

In this example, beliefs do not converge quickly to reflect the optimal solution (figure 2*b*). Indeed, even after 30 trials, it is still unclear which items are in the optimal solution, and hence, which securities will pay $ 1. To make matters worse, beliefs evolve arbitrarily, that is, they move from high to low and vice versa even in later trials. This behaviour of beliefs is in stark contrast with the previous case, where uncertainty was reduced steadily by random sampling (figure 2*a*).

Since there are only a finite number of possible capacity-filled knapsacks, our decision-maker should eventually come across the optimal solution. This will happen when, just by chance, the best knapsack is sampled. But this will take time. In the present situation, there are (only!) 82 possible capacity-filled knapsacks, so the chance of drawing the best knapsack in any trial equals 1/82; this implies, among others, that the chance that the algorithm finds the optimal knapsack within 30 trials is only about 5%.

To demonstrate how ineffective (random) sampling is in this example, notice that it takes approximately 400 trials to increase to 95% the chance of coming across the optimal knapsack. But there are only 82 capacity-filled knapsacks, so in this case it would have been much more effective to list all 82 possibilities and pick the optimum. In other words, brute-force search would find the optimal solution in 82 steps, while the sampling approach will only find it with 95% probability even after five times as many steps (samples). This shows that (random) sampling in this example is not an effective way to reduce uncertainty.

Indeed, it has been shown that in tasks like the one in this example, participants do not use random sampling to find the optimal solution but instead use more effective algorithms such as the greedy algorithm or branch-and-bound. In an experiment where participants were paid to solve different instances of the 0–1 knapsack problem, they performed far better than if they had used random sampling of potential solutions [35]. Their initial choices reflected the greedy algorithm. However, greedy algorithms often do not lead to the optimal solution. Many participants deviated from greedy algorithms later in the trials, in ways that look like branch-and-bound algorithms, specialized algorithms to solve the knapsack problem.

A critical issue arises in such situations in which the decision-maker acts in a way that is not optimal relative to the Savage framework: it is not always possible to define state spaces and beliefs that, on the one hand, accurately represent choices and learning of the agent and, on the other hand, obey the Savage axioms [16].

Our second example indicates that the Savage framework is only effective in representing a particular type of uncertainty (lack of knowledge), namely, uncertainty that can effectively be represented by probabilities. It assumes that uncertainty is entirely due to randomness, and that uncertainty is reduced through statistical inference, that is, inference that is only concerned with randomness, using the rules of probability theory [3]. Many decision tasks, however, involve a kind of uncertainty—uncertainty due to computational complexity—for which such a representation is not appropriate. Here, other forms of inference are often more effective than statistical inference, for example, deduction. Indeed, algorithms designed to solve the 0–1 knapsack problem exploit the structure of the problem and typically only explore a small proportion of the search space to generate enough information to solve an instance. Importantly, people seem to reduce uncertainty in problems like the knapsack problem using strategies that much closer resemble effective computer algorithms than statistical inference based on random sampling [34,35].

The inefficiency of random search has been analyzed theoretically using so-called randomized algorithms. Such algorithms inject randomness in the computation of the solution, either at the level of the input or at the level of the algorithm itself [57]. As a result, different executions of such algorithms may result in different solutions and different execution times [58]. An algorithm may execute in polynomial time but may return an output whose value is a random variable (‘Monte Carlo’ algorithms). Alternatively, the algorithm may always return the optimal solution but have an execution time that is a random variable (‘Las Vegas’ algorithms). Currently, it is unknown if there exists a polynomial-time randomized algorithm that can compute an optimal solution for an **NP-hard** problem [59]. Thus, at present, randomized algorithms do not overcome the challenges posed by computational complexity. In particular, they do not provide a generic approach to overcoming the computational resource requirements of decision tasks.

### (c) Implications for modelling of decisions

As we pointed out in the Introduction, the Savage framework has not only been the most popular framework for modelling of decisions under uncertainty. It has also been suggested to be a generic framework that can capture any decision situation. Indeed, the Savage framework has inspired the development of many new frameworks that capture one or more situations in which the Savage axioms are violated, such as the Allais and Ellsberg paradoxes. Such frameworks include Gilboa–Schmeidler preferences [60], disappointment version [61], cumulative prospect theory [62] and rank-dependent expected utility [63]. However, all of these theories assume that the agent behaves optimally, as if it maximized a weighted average of utilities over states.

In this section, we discussed two severe weaknesses of the Savage framework. Firstly, we used tools from computational complexity theory to show that the Savage framework is computationally intractable, rendering its core axioms implausible. Secondly, we showed that the Savage framework is ineffective in representing decision tasks with high degrees of computational complexity.

To address the first of the above issues, future models of decision-making should take into account the computational complexity of decision tasks. For such models to be plausible, the computational requirements necessary to solve a particular decision task should not exceed the decision-maker’s computational capacities. Future work will need to (i) characterize the computational resource requirements of decision tasks encountered in ecologically relevant settings and (ii) characterize the computational resources available to decision-makers in such settings [30,34,64].

The latter is complicated by the fact that the brain likely allocates computational resources dynamically. At this point, it is an open question which mechanism the brain uses for the allocation of computational (cognitive) resources, a problem referred to as *cognitive control* or *meta-decision-making* [34,65,66]. Most frameworks of cognitive control are based on the concept of optimization: decision-makers optimally allocate resources to tasks, trading off (expected) rewards gained from the task and costs of the resources deployed in the task [66,67]. These frameworks suffer from the same weaknesses as the Savage framework: many of those models imply computational problems with high degrees of computational complexity. Therefore, many of those models are not plausible from a computational perspective [34].

To address the second issue above—ineffectiveness of the Savage framework in representing certain types of uncertainty—frameworks will need to be developed that allow more general representations of knowledge (and learning) than those afforded by probabilities. Such frameworks should include conceptual and computational primitives such as representations of objects, structured, algebraic representations, operations over variables, causality and reasoning with counterfactuals, among others [21,68,69].

The latter is particularly relevant for recently proposed models of cognition based on the Bayesian Brain Hypothesis [70]. In those models, all knowledge is represented by—that is, reduced to—probabilities and new information is incorporated using Bayes’ Law. Both issues raised earlier in this section in relation to the Savage framework also apply to those models: they are generally computationally intractable and ineffective in representing knowledge (and its reduction). Indeed, it has recently been shown that several components of those models have degrees of computational complexity even higher than that of the knapsack problem discussed earlier [51]. It is therefore unlikely that these models provide an accurate account of cognition, including decision-making [21].

## 7. Conclusion

Modern theories of decision-making typically model uncertainty about decision options using the tools of probability theory, as exemplified by the Savage framework, the most popular framework in decision-making research [1]. In this article, we used concepts from computational complexity theory to argue that in many situations, the Savage framework is computationally intractable and, in situations in which computational complexity is high, ineffective in representing uncertainty. We also discussed new empirical evidence supporting our claims, casting further doubt on the plausibility of the framework’s axioms.

The empirical evidence, in particular, calls for new theories of decision-making that are plausible from a computational as well as biological perspective. This means that computational resource requirements implied by such theories need to be within the resources available to decision-makers. Computational complexity theory provides a candidate framework for quantifying computational resource requirements. The endeavour will be complicated by the fact that allocation of cognitive resources in the brain is likely dynamic, dependent on motivation, physiological state and other context-dependent factors.

Some immediate questions arise for the future study of decision-making. On the one hand, it will be important to characterize the computational complexity of decisions encountered in every-day life, that is, the distribution of real-world instances of key computational problems encountered in ecologically relevant settings. On the other hand, it should be investigated which strategies people use to make complex decisions, how they adapt to varying levels of computational complexity and how resources are allocated to decision-making.

More accurate theories of decision-making are desirable not only from a scientific perspective but also from the perspective of public policy, including regulation. In some domains such as financial markets, regulation of goods and services is premised on rational-actor models. That is, consumers are assumed to make rational choices in the sense of Savage—despite the fact many decisions consumers face have high degrees of computational complexity. Those decisions include savings and investment decisions or the selection of insurance contracts. In the light of the evidence presented in this article, this assumption is clearly implausible. Computationally (and biologically) plausible models of decision-making will improve the design of products, services and markets as well as their regulation, with the potential for substantial gains in welfare.

## Data accessibility

This article has no additional data.
